# Dataset of the *Botrytis cinerea* phosphoproteome induced by different plant-based elicitors

**DOI:** 10.1016/j.dib.2016.04.039

**Published:** 2016-04-22

**Authors:** Eva Liñeiro, Cristina Chiva, Jesús M. Cantoral, Eduard Sabido, Francisco Javier Fernández-Acero

**Affiliations:** a Andalusian Center for Grape and Grapevine Research, CeiA3, Marine and Environmental Sciences Faculty, University of Cadiz, Puerto Real, 11510 Cádiz, Spain; b Proteomics Unit, Centre for Genomic Regulation (CRG), Dr. Aiguader, 88, 08003 Barcelona, Spain; c Proteomics Unit, Universitat Pompeu Fabra (UPF), Dr. Aiguader, 88, 08003 Barcelona, Spain

**Keywords:** TCW, Tomate Cell Walls, Phosphoproteome, *Botrytis cinerea*, Mass spectrometry, Proteomics, Plant-like elicitors, Fungal pathogen

## Abstract

Phosphorylation is one of the main post-translational modification (PTM) involved in signaling network in the ascomycete *Botrytis cinerea*, one of the most relevant phytopathogenic fungus. The data presented in this article provided a differential mass spectrometry-based analysis of the phosphoproteome of *B. cinerea* under two different phenotypical conditions induced by the use of two different elicitors: glucose and deproteinized Tomate Cell Walls (TCW). A total 1138 and 733 phosphoproteins were identified for glucose and TCW culture conditions respectively. Raw data are deposited at the ProteomeXchange Consortium via the PRIDE partner repository with the data set identifier (PRIDE: http://www.ebi.ac.uk/pride/archive/projects/PXD003099). Further interpretation and discussion of these data are provided in our research article entitled “Phosphoproteome analysis of *B.cinerea* in response to different plant-based elicitors” (Liñeiro et al., 2016) [1].

**Specifications Table**

**Table:** 

Subject area	*Biology*
More specific subject area	*Microbiology, fungi, phytopathogens*
Type of data	*Table*
How data was acquired	LC-MS/MS LTQ-Orbitrap Velos Pro (Thermo Fisher Scientific, San Jose, CA) coupled to a nano-LC (Proxeon, Odense, Denmark)
Data format	*Raw, analyzed*
Experimental factors	Isolation and characterization of *Botrytis cinerea* phosphoproteins induced with Glucose (constitutive) or Deproteinized tomato cell walls (induction) as a sole carbon sources.
Experimental features	*B. cinerea was cultured in the presence of Glucose or deproteinized TCW as a sole carbon sources. Total proteins were extracted, trypsin digested and the enriched in phosphopeptides by TiO2 micro-columns. Phosphopeptides were identified by MS/MS*
Data source location	*Centre de Regulació Genòmica 08003 Barcelona, España*
Data accessibility	Data is within this article and at the ProteomeXchange Consortium^31^ via the PRIDE accession number PRIDE: http://www.ebi.ac.uk/pride/archive/projects/PXD003099)

**Value of the data**

• The characterization of the *B. cinerea* phosphoproteome under different induction conditions is the first comparative phosphoproteomic data set in this model phytopathogenic fungus.
• The identified phosphopeptides contribute to expand the map of known phosphoproteins in this pathogen.
• This data set defines the role of the phosphoproteins involved in the signaling pathways.
• It enables the advance in the design of novel strategies against the fungi.

## 1 Data

This proteomics dataset comprises the total set of phosphopeptides (Supplementary Table 1) and phosphoproteins identified (Supplementary Table 2) from a comparative analysis of the fungal pathogen *Botrytis cinerea* growth under two different phenotypical conditions induced by use of two different carbon sources: Glucose (GLU) and deproteinized tomato cell wall (TCW), which have been previously proved efficient to induce different fungal responses. Supplementary Table 3 shown the identified proteins divided in three different groups according to their absence/presence in the two assayed conditions: (i) phosphoproteins identified in both assayed conditions (Common phosphoproteins), (ii) phosphoproteins identified only under induction with GLU and (iii) phosphoproteins identified only under induction with TCW. Raw LC-MS/MS files are also available via ProteomeXchange Consortium^31^ via the PRIDE accession number (PRIDE: http://www.ebi.ac.uk/pride/archive/projects/PXD003099).

## 2 Experimental design, materials and methods

### 2.1 *B.cinerea* culture

*B. cinerea* B05.10 strain (provided by Dr Paul Tudzynski from the University of Münster, Germany) was maintain in conidial stock suspension as previously reported in González-Rodriguez et al., 2015 [2]. Conidial suspension were inoculated to a final concentration of 5·10^4^ conidia/mL in 250 mL of Minimal salt medium (50 mM NH4Cl, 7.3 mM KH2PO4, 4.2 mM MgSO4, 6.7 mM KCl, 0.07 mM FeSO4) supplemented with 1% of a sole carbon source: glucose (GLU) (Panreac, Spain) or tomato cell wall (TCW) obtained from tomato fruits (*Lycopersicon esculentum* cv bola) of commercial maturity and deproteinized as previously reported [3]. After 5 days of culture in an orbital shaker at 180 rpm and at 22 °C mycelia were harvest by filtration in a 30-µm nylon filter (Sefar Nytal, Switzerland) and stored at −80 °C until its use for total protein extraction. Four independent biological replicas were cultured by assayed carbon source.

### 2.2 Protein extraction and trypsin digestion

For each biological replica, mycelium was ground into a fine powder using a mortar and pestle in presence of liquid nitrogen. A phenol-based method previously reported [4] was applied for the extraction of mycelial proteins. Total extracted proteins were resuspended in 300 µL of dissolving buffer (6 M urea, 0.2 M ammonium bicarbonate). For protein digestion, 500 µg of each sample were reduced with dithiothreitol (10 mM, 37 °C, 60 min), and alkylated with iodoacetamide (20 mM, 25 105 °C, 30 min). Samples were diluted up to 2 M urea, digested overnight with Lys-C at 37 °C, and then diluted again 2-fold prior being digested overnight with trypsin at 37 °C. Crude peptide mixtures were desalted using a MacroSpin Column C18 column (The Nest Group, Inc, Southborough, MA). Protein quantification was conducted by Qubit 2.0 Fluorometer sistem (Invitrogen, USA).

### 2.3 Phosphopeptide enrichment

Phosphopeptide enrichment was conducted from 250 μg of each digested sample by titanium dioxide (TiO_2_) micro-columns. TiO2 matrix were prepared in gel loading tips (0.5 mg) and equilibrated with loading buffer (80% ACN in water+6% TFA). Samples were loaded in loading buffer and the columns were washed with 80% ACN in water+0.1% TFA. Finally, phosphopeptides were eluted with 30 µL of elution buffer (5% NH_3_ in water) into a 1.5-mL tube containing 30 µL of 20% of formic acid in water. Crude phosphopeptide mixtures were desalted using a MacroSpin Column C18 column (The Nest Group, Inc, Southborough, MA).

### 2.4 LC-MS/MS and data analysis

The enriched phosphopeptide mixture was analyzed using a LTQ-Orbitrap Velos Pro mass spectrometer (Thermo Fisher Scientific, San Jose, CA) coupled to a nano-LC (Proxeon, Odense, Denmark) equipped with a reversed-phase chromatography column (25 cm, inner diameter of 75 μm, packed with 1.9 μm C18 particles; Nikkyo Technos, Japan). A gradient of 3 to 35% acetonitrile with 0.1% formic acid in 360 min at a flow of 250 µL/min was used. The Orbitrap Velos was operated in positive ion mode with nanospray voltage set at 2.2 kV and source temperature at 325 °C. The instrument was externally calibrated using Ultramark 1621 for the FT mass analyzer. An internal calibration was performed using the background polysiloxane ion signal at m/z 445.120025 as the calibrant. The instrument was operated in data-dependent mode. In all experiments, full MS scans were acquired over a mass range of m/z 350–2000 with detection in the Orbitrap mass analyzer at a resolution setting of 60,000. For each MS scan, the twenty most intense ions with multiple charged ions above a threshold ion count of 5000 were fragmented using collision-induced dissociation with multistage activation (activation of neutral losses of 98, 65.4, 49, 32.7) at a normalized collision energy of 35% in the LTQ linear ion trap. All data were acquired with Xcalibur v2.2 (Thermo Fisher Scientific, United States). 106 Protein identification was performed with the Proteome Discoverer software suite v.1.4.0.288 (Thermo Fisher Scientific, United States) using MASCOT v2.3 (Matrix Science, United Kingdom) as search engine. A *B. cinerea* NCBI database that included the most common contaminants was used, and carbamidomethylation for cysteines was set as fixed modification whereas acetylation in protein N-terminal, phosphorylation in Ser, Thr and Tyr and oxidation of methionine were set as variable modifications. Peptide tolerance was set at 7 ppm in MS and at 0.5 Da in MS/MS mode. The maximum number of missed cleavages was set at 3, and peptides were filtered based on an false discovery rate (FDR) lower than 1%. For comparative analysis only those proteins identified in at least 3 of the 4 biological samples run per assayed condition were consider.
