# Supplementary material for: Dataset of the Botrytis cinerea phosphoproteome induced by different plant-based elicitors
Source: Data Brief. 2016 Apr 22;7:1447–50. doi: 10.1016/j.dib.2016.04.039 (PMC5063813; doi:10.1016/j.dib.2016.04.039)
Supplement: Supplementary file 1 — Supplementary material [file mmc1.docx]

**Conflict of interest:**

The authors have declared no conflict of interest.
